# Five-Year Follow-Up of Cured HCV Patients under Real-World Interferon-Free Therapy

**DOI:** 10.3390/cancers13153694

**Published:** 2021-07-22

**Authors:** Robert Flisiak, Dorota Zarębska-Michaluk, Ewa Janczewska, Tadeusz Łapiński, Magdalena Rogalska, Ewa Karpińska, Tomasz Mikuła, Beata Bolewska, Jolanta Białkowska, Katarzyna Flejscher-Stępniewska, Krzysztof Tomasiewicz, Kornelia Karwowska, Monika Pazgan-Simon, Anna Piekarska, Hanna Berak, Olga Tronina, Aleksander Garlicki, Jerzy Jaroszewicz

**Affiliations:** 1 Department of Infectious Diseases and Hepatology, Medical University of Bialystok, 15-540 Bialystok, Poland; pmagdar@gmail.com; 2 Department of Infectious Diseases, Jan Kochanowski University, 25-369 Kielce, Poland; dorota1010@tlen.pl; 3 Department of Basic Medical Sciences, Faculty of Health Sciences in Bytom, Medical University of Silesia, 41-902 Bytom, Poland; e.janczewska@poczta.fm; 4 Department of Infectious Diseases, Hepatology and Liver Transplantation, Pomeranian Medical University, 70-204 Szczecin, Poland; ewakarpinska@interia.pl; 5 Department of Infectious and Tropical Disease and Hepatology, Medical University of Warsaw, 02-091 Warsaw, Poland; tomasz.mikula6@wp.pl; 6 Department of Infectious Diseases, Poznan University of Medical Sciences, 61-701 Poznan, Poland; bbolewska@ump.edu.pl; 7 Department of Infectious and Liver Diseases, Medical University of Lodz, 90-419 Lodz, Poland; jolanta.e.bialkowska@gmail.com; 8 Department of Infectious Diseases, Liver Diseases and Immune Deficiencies, Wroclaw Medical University, 50-367 Wroclaw, Poland; kasia.fleischer@wp.pl; 9 Department of Infectious Diseases and Hepatology, Medical University of Lublin, 20-059 Lublin, Poland; tomaskdr@poczta.fm; 10 Department of Infectious Diseases and Hepatology, Collegium Medicum, Nicolaus Copernicus University, 87-030 Bydgoszcz, Poland; kornelia.karwowska@cm.umk.pl; 11 Department of Infectious Diseases and Hepatology, Wroclaw Medical University, 50-367 Wroclaw, Poland; monikapazgansimon@gmail.com; 12 Department of Infectious Diseases and Hepatology, Medical University of Lodz, 90-419 Lodz, Poland; annapiekar@gmail.com; 13 Daily Unit, Hospital of Infectious Diseases in Warsaw, 01-201 Warsaw, Poland; hberak@zakazny.pl; 14 Department of Transplantation Medicine, Nephrology and Internal Medicine, Medical University of Warsaw, 02-091 Warsaw, Poland; olgatronina@wp.pl; 15 Department of Infectious and Tropical Diseases, Jagiellonian University Medical College, 31-008 Krakow, Poland; agarlicki@gmail.com; 16 Department of Infectious Diseases and Hepatology, Medical University of Silesia, 40-055 Katowice, Poland; jerzy.jr@gmail.com

**Keywords:** viral hepatitis C, liver cirrhosis, hepatocellular carcinoma, sustained virologic response, long-term follow-up, therapy

## Abstract

**Simple Summary:**

Hepatitis C virus (HCV) is the major factor responsible for hepatocellular carcinoma (HCC). Currently available treatments for HCV infection are short, simple, effective and safe. Long-term monitoring of patients is essential to demonstrate the efficacy of antiviral therapy, including the risk of HCC development. Since highly effective treatment options only became available in the middle of the past decade, we evaluated patients treated during this period five years after treatment. We have shown that the risk of death due to HCC as well as death due to HCV persists through 5 years of follow-up after successful treatment. Therefore, longer follow-up is necessary to assess the long-term risk of developing HCC, especially in patients with cirrhosis.

**Abstract:**

(1) Background: Treatment of hepatitis C virus (HCV) infections with direct-acting antivirals (DAA) has demonstrated high efficacy and an excellent safety profile. The cured patients showed a sustained virological response and improved liver function, but also a continued risk of hepatocellular carcinoma (HCC) during the 2–3 years of follow-up after treatment; (2) Methods: A total of 192 patients out of 209 of the primary AMBER study were analyzed five years after treatment with ombitasvir/paritaprevir/ritonavir with or without dasabuvir and with or without ribavirin. Results: We confirmed that HCV clearance after DAA treatment is stable regardless of baseline liver fibrosis. We found that sustained virologic response is associated with a gradual but significant reduction in liver stiffness over 5 years. Liver function improved during the first 2 years of follow-up and remained stable thereafter. The risk of death due to HCC as well as death due to HCV persists through 5 years of follow-up after successful DAA treatment. However, in non-cirrhotic patients, it appears to clear up 3 years after treatment; (3) Conclusions: Monitoring for more than 5 years after curing HCV infection is necessary to assess the long-term risk of possible development of HCC, especially in patients with cirrhosis of the liver.

## 1. Introduction

Highly effective and safe direct acting antivirals (DAAs) have changed the global epidemiological situation of hepatitis C virus (HCV) infection. New treatment regimens that have emerged in Europe since 2014 have eliminated treatment waiting lists and cured those who had not responded to previous interferon-based therapies [1,2,3]. However, most important was the potential for therapeutic success in patients with advanced liver disease that could prevent its progression, including the development of hepatocellular carcinoma (HCC). DAAs significantly improved the efficacy and safety of HCV treatment compared to previous interferon-based regimens [4]. One of the first available treatment options not requiring interferon included the NS5A inhibitor ombitasvir (OBV), the NS3/4A protease inhibitor paritaprevir (PTV) ritonavir-boosted (r), and the NS5B polymerase inhibitor dasabuvir (DSV), was used with or without ribavirin (RBV), and was approved for the treatment of HCV-infected patients at the end of 2014 [5,6,7].

Due to the lack of real-world experience (RWE) data, a multicentre, open-label AMBER study, initiated by investigators, was conducted in 2014–2015 to investigate the efficacy and safety of OBV/PTV/r ± DSV ± RBV. AMBER was the first RWE study in the world with this therapeutic regimen to demonstrate a high SVR rate and an excellent safety profile, even in patients with cirrhosis, a history of interferon-based therapy failure, and after liver transplantation [8]. Further studies published by other authors confirmed our findings from the 12-week post-treatment assessment [9,10,11,12,13,14]. A further two-year follow-up of patients participating in the AMBER program confirmed the persistence of the virological response, which was accompanied by a significant improvement in the main functional indicators including, in particular, a reduction in liver stiffness (LS). However, effective therapy did not prevent liver decompensation, HCC, or cirrhosis-related deaths, which supported the need for longer monitoring [15].

The purpose of this study is to evaluate the persistence of the virologic response, possible changes in liver function, liver stiffness and risk of HCC five years after treatment in the AMBER study.

## 2. Materials and Methods

Data for the current assessment were collected from 15 hepatology centers in Poland that obtained early access to OBV/PTV/r ± DSV ± RBV therapy under the named patient programme, which started before approval of this medication by the European Medicines Agency in 2014. Each center obtained approval from the local ethics committee for experimental therapy prior to enrollment to the primary AMBER study, and written informed consent was obtained from each patient before the treatment initiation. The current study was carried out as a retrospective, observational, non-interventional analysis, and all data were collected as a regular medical standard procedures.

### 2.1. Study Population and Design

Patients included in the pivotal AMBER study were adults with chronic genotype 1 or 4 HCV infection who completed OBV/PTV/r ± DSV ± RBV in the AMBER study, and their data were available 5 years after the end of therapy [8]. In the meantime, patients included in the AMBER study were monitored 2 years after the end of treatment (2yFU), and the data from this analysis were published in 2018 [15]. The status of patients was reassessed 5 years after the end of treatment (5yFU) with available data within the acceptable time window of ±6 months. Of the 209 participants in the pivotal study, 17 patients did not respond to telephone or letter invitations and family members were unable to provide any information regarding the patient’s condition, so they were considered lost for follow-up after 5 years. The baseline characteristics of the patients available for evaluation at 5yFU are shown in Table 1. Patients were assessed for medical events including death, ascites, encephalopathy, upper gastrointestinal bleeding, diagnosis of HCC, and liver transplantation. Liver ultrasound data were collected 12 weeks after EOT, and then 2yFU and 5yFU, with cirrhosis patients being tested every 3–6 months in accordance with national recommendations [16]. Survival information was collected directly from patients or family members in the event of death. The relationship between death and HCV infection was assessed by a physician based on the obtained information. HCV-related death was considered to have occurred as a result of previous HCV infection, which in practice meant the progression of liver disease, development of HCC and liver decompensation despite successful elimination of HCV infection. HCV RNA was determined at 5yFU in 148 patients to assess the long-term persistence of the virological response. Other parameters that were compared between baseline, end of treatment (EOT), 2yFU, and 5yFU included liver stiffness (LS), bilirubin, albumin, and creatinine, which allowed the calculation of the Child–Pugh score and MELD (Model for End-Stage Liver Disease). LS was measured with a non-invasive, real-time, quantitative elastography technique, applied in particular centers with either transient elastography (TE) using FibroScan^®^ equipment (Echosens™, Paris, France) or shear wave elastography (SWE) using Aixplorer^®^ equipment (Super Sonic Imagine™, Aix-en-Provence, France).

### 2.2. Statistical Analysis

The results are expressed as mean ± standard deviation (SD) or *n* (%). *p* values of <0.05 were considered to be statistically significant. The significance of difference was calculated by the Fisher’s exact test for nominal variables and by Wilcoxon matched paired test for continuous variables. Survival analyses were performed by log-rank (Mantel–Cox). T-test supported by the Mantel–Haenszel hazard ratio (MH HR) and its 95% confidence interval as the effect size measure were depicted as Kaplan–Meier (KM) plots. Statistical analyses were performed by use of GraphPad Prism 5.1 (GraphPad Software, Inc., La Jolla, CA, USA).

## 3. Results

A total of 192 patients out of 209 from the original study were included in the current analysis. As shown in Table 1, most of them were infected with HCV genotype 1b (85.4%), prior interferon-based therapy was unsuccessful (66.7%), and cirrhosis was diagnosed at baseline (57.3%). Reports of HCV RNA tests at 5yFU were available in 148 patients and all were undetectable for HCV RNA.

As shown in Table 2, significantly higher annual death rates were observed in the 2- to 5-year follow-up period than in the first 2 years after treatment. This was also true for HCV-related deaths. Deaths related to HCV or HCC in the last 2 years were recorded only in patients with baseline cirrhosis (Table 3). Signs of liver decompensation appeared less frequently in the last 3 years of follow-up, but the difference was not statistically significant. Despite the reduced incidence of HCC after the second year of follow-up, there was no statistically significant difference from the initial follow-up period. There was no difference regarding frequency of liver transplantation (Table 2).

**Figure 1:**
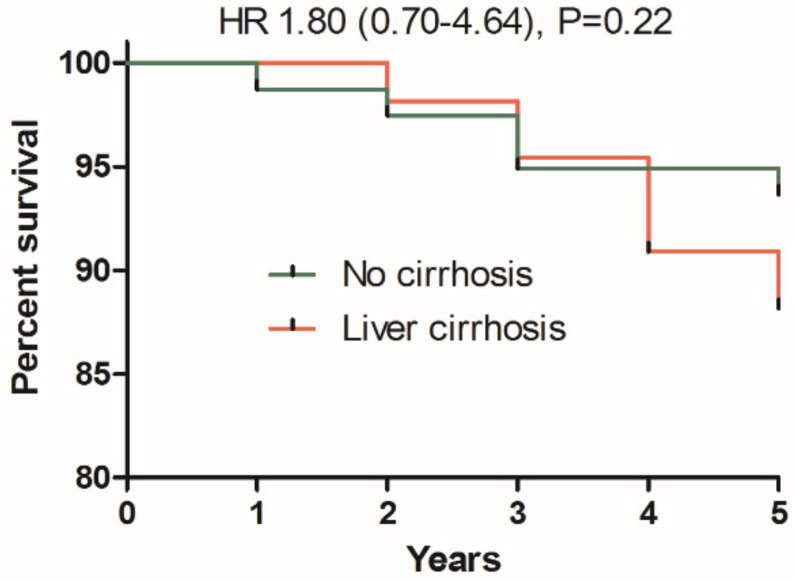
Kaplan–Meier plots of patients survival depending on presence of cirrhosis at the baseline.

**Figure 2:**
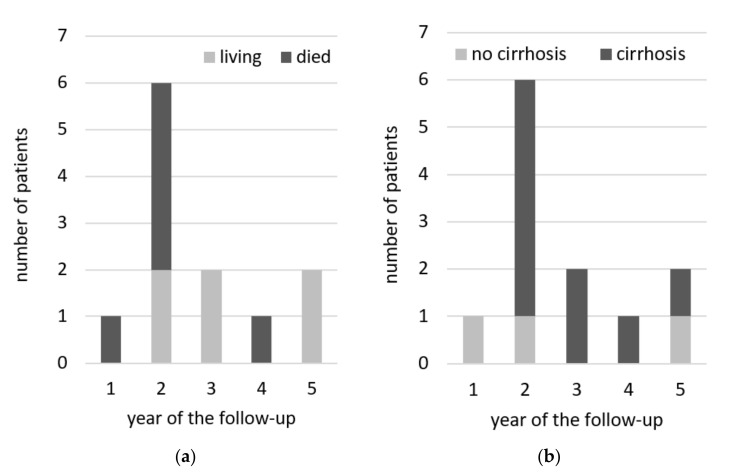
Hepatocellular carcinoma diagnosed in particular years of the follow-up, with regards to patients survival (**a**) and presence of cirrhosis at the baseline (**b**).

As shown in Figure 1 there was no significant difference in mortality risk depending on the presence of cirrhosis at the baseline. However, there was a tendency of decreased survival in cirrhotics in the last two years of follow-up that need to be monitored in the future (Figure 1). Among 12 diagnosed HCC cases, three had no cirrhosis at baseline, and one was diagnosed only at year 5 of follow-up (Figure 2). There were no chronic comorbidities noted in HCC patients, except single cases of Hodgkin lymphoma and epilepsy.

Among 14 deceased patients in whom the treating physician has recognized death as HCV-related, only 3 were not diagnosed with cirrhosis before treatment, but 2 of these 3 had advanced fibrosis (F3). Analysis of the MELD and Child–Pugh scores indicates progression of liver disease with incidents of decompensation during post-treatment follow-up in 9 of 11 patients with available data from this group.

A statistically significant reduction in bilirubin, INR, MELD and Child–Pugh scores, as well as an increase in albumin, was observed when comparing the results between EOT and 2yFU. However, in the following 3 years the changes were not significant (Table 4). BMI values increased significantly in the 2yFU period, and in the next 3 years they also showed a statistically significant increase. After 5 years of follow-up, the normal concentration of albumin was found in 95%, bilirubin in 80%, and INR in 88% of the subjects.

**Figure 3:**
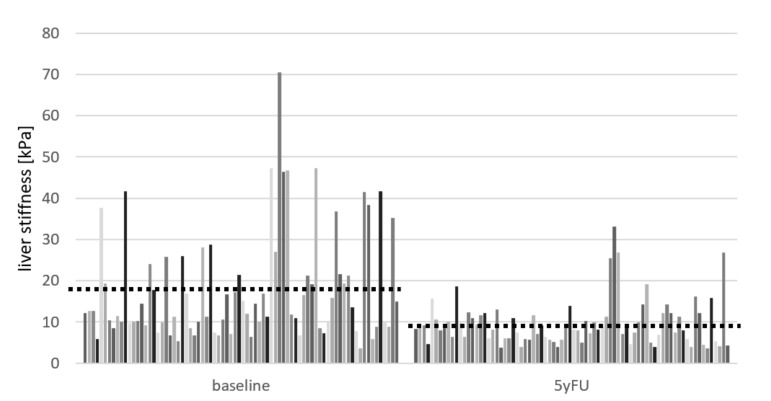
Individual values (bars) and mean (dashed horizontal line) of the liver stiffness (kPa) measured at the baseline (before start of the treatment) and 5yFU (5 years following end of treatment) in patients with available paired data.

**Figure 4:**
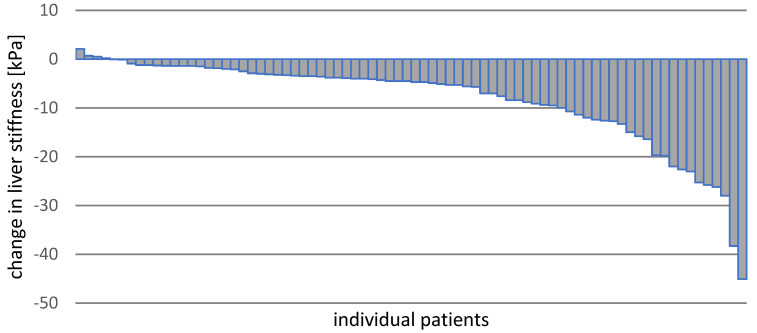
Changes in the liver stiffness between baseline and 5yFU in patients with available paired data.

Liver stiffness results at baseline, EOT, 2yFU, and 5yFU were available in 78 patients. LS decreased significantly from 17.9 ± 13.0 kPa at baseline to 9.65 ± 5.59 kPa at 5yFU, and the reduction in stiffness was also significant between the individual control time points (Table 4, Figure 3). A reduction in individual LS values during the five years of follow-up after treatment was observed in 74 (95%) patients, and 57 (73%) showed a reduction in stiffness of at least 3 kPa (Figure 4). The increase in LS during 5yFU was demonstrated only in 4 patients and did not exceed 2.1 kPa (Figure 4).

## 4. Discussion

To the best of our knowledge, the present study covers the most prolonged follow-up in a DAA-cured population, which is largely composed of patients with compensated liver cirrhosis.

The HCV clearance is a primary endpoint of the antiviral therapy regardless of the advancement of liver disease. Our analysis confirmed the durability of the virologic response following the DAA therapy across all liver fibrosis stages. Long-term persistence of viral clearance was documented in patients with advanced liver disease in Austrian and Portuguese RWE cohorts treated with DAA, but the observation period was much shorter than ours [17,18]. Both studies analyzed patients predominantly treated with IFN-free, SOF-based regimens, 87% and 95%, and reported durability of viral eradication over a follow-up period of 65.6 weeks and 28 months, respectively.

Of the two ongoing clinical trials designed to evaluate long-term liver disease progression and clinical outcomes for up to 5 years post-treatment of OBV/PTV/r ± DSV ± RBV, in 2211 patients infected with GT1, the interim results after 3 years are available [19].

However, the main objective of our analysis was to assess how this durable HCV eradication affects hepatic function parameters, liver stiffness, and the risk of liver disease-related complications.

It is worth noting that while the SVR is a universal milestone of HCV treatment, the remaining goals vary depending on the initial liver fibrosis. The main benefit of the viral clearance for patients with mild to moderate liver disease is the prevention of fibrosis progression, the improvement of the quality of life, and elimination of the risk of HCV transmission, so non-cirrhotic patients are usually discharged from further follow-up and available data on the post-clearance course of the disease in this population are limited. Conversely, the patients with liver cirrhosis constituting the majority in the analyzed population are considered to be the most at risk of serious liver complications such as hepatic decompensation in the form of ascites and encephalopathy, the upper gastrointestinal bleeding as a consequence of portal hypertension, and hepatocellular carcinoma. We have shown that the overall and annual prevalence of these severe medical events was lower from the third year of follow-up than during the first 2 years, which means less risk of complications over time, and our findings supported the results of other studies [19,20,21,22,23,24,25]. However, none of these studies covered such a long period of 5 years follow-up.

The mean values of selected liver function parameters, such as bilirubin level, albumin concentration, and INR, corresponding to the Child–Pugh and MELD scores, changed significantly during the first two years of follow-up and remained stable thereafter. The increase of albumin level after OBV/PTV/r ± DSV ± RBV therapy was documented in non-cirrhotics and compensated cirrhotics in the TOPAZ-I and TOPAZ-II trials after 2 and 3 years of FU, and the improvement was more pronounced in patients with liver cirrhosis, which was earlier noticed after 2 years’ follow-up of patients included in our study [15,19]. Our current results are similar to those obtained in the real-world TARGET study; however, the differences in the baseline characteristics of the studied populations should be noted [26]. One-fourth of the cohort of 642 patients with advanced/decompensated HCV-related cirrhosis achieved a significant improvement in liver function parameters in the short-term follow-up, whereas over a median observation of 4 years after DAA therapy, the mean changes in bilirubin, albumin, and MELD values were marginal.

A different trend within the current study was observed when analyzing the changes in LS. While the liver function parameters improved significantly during the short-term after DAA treatment, the reduction of LS was observed during the whole follow-up period. In both evaluated time intervals, between EOT and 2yFU and between 2yFU and 5yFU, the difference was statistically significant. Multi-annual observations on the impact of HCV cure on the reduction of LS focus mainly on patients with liver cirrhosis and come from studies carried out in the era of IFN-based treatment, while available reports on DAA therapy refer to a shorter follow-up period. As we have shown in our previous analysis, LS reduction between EOT and 2 years’ follow-up was significantly bigger in cirrhotics than non-cirrhotics, and the same tendency we observed regarding albumin and bilirubin concentrations, as well as Child–Pugh score [15]. Fifty-four cirrhotic patients were investigated by Knop et al. [27], and significant improvement of LS was documented between baseline and EOT and from baseline to FU24, whereas the difference between EOT and FU24 was less pronounced. The significant regression of mean LS was documented in a Spanish cohort of 554 patients with compensated liver cirrhosis from a baseline of 20.2 kPa to 13.9 kPa after one year of DAA treatment [25]. The significant reduction of LS by 15% between EOT and 1-year FU in 71 patients with advanced liver disease treated successfully with IFN-free therapy was demonstrated by Laursen et al. [28]. The highest rate of reduction in elastometry values during treatment and the first months following SVR, documented in numerous short follow-up studies, is currently confirmed also in our long-term analysis. This effect should be considered to be related rather to a decrease of the necroinflammatory activity, whereas the long-term decline is influenced by the real fibrosis regression [20,29,30].

Among the benefits of HCV cure, the medium- and long-term studies documented the improvement of survival in patients with compensated liver cirrhosis [21,31,32,33,34]. The HCV clearance was demonstrated to be an independent factor associated with the reduction of risk of both liver-related and non-liver-related mortality [19,33,35]. The current study reported a total of 14 deaths classified as related to HCV or HCC, corresponding to an annual rate of 0.7 in the first 2 years and 1.9 between 2yFU and 5yFU. Deaths related to HCV or HCC in the last 2 years of observation were reported only in patients with baseline cirrhosis. A total of 12 cases of HCC were diagnosed during the whole duration of follow-up, 7 within the first 2, and 5 in the subsequent 3 years with annual rates of 1.7 and 0.9, respectively. Three of 7 HCC diagnosed between EOT and 2yFU were recurrent, while between 2yFU and 5yFU, only de novo HCC cases were reported.

At the beginning of the IFN-free era, warnings about the possible association between the DAA therapy and higher incidence of de novo and recurrent primary liver cancer were raised based on the previously published reports documenting the greater crude risk of HCC in IFN-free compared to IFN-containing recipients [36,37]. However, after adjusting for baseline confounders, this relation was not confirmed, and most subsequent analyses demonstrated the positive impact of SVR on this endpoint of DAA treatment. In the longest published analysis to date from the clinical trial, the proportion of patients with compensated cirrhosis diagnosed with HCC up to 3 years after treatment with OBV/PTV/r ± DSV ± RBV was as low as 1.4% [19]. Our observations on the reduced risk of liver cancer in cirrhotic patients following HCV clearance are consistent with results coming from numerous RWE cohorts and meta-analysis of earlier studies, but we were able to demonstrate this DAA effect during the longest follow-up period [21,38,39,40,41,42]. It should be emphasized that despite the dominance of SOF-based regimens in most studies available in large patient populations, the reduction in the risk of HCC after DAA therapy was observed regardless of the therapeutic regimen used [18,40,43,44].

Although hepatocellular carcinoma was diagnosed less frequently in the period between 2yFU and 5yFU than shortly after treatment, it should be emphasized that even 5 years after completing effective DAA therapy, patients with pre-existing advanced liver fibrosis and cirrhosis are at substantial risk of liver cancer. Our study shows that DAA-induced HCV clearance does not eliminate the risk of de novo HCC entirely even in non-cirrhotic patients, supporting the results of other analyses and recommendations on ongoing cancer surveillance in this population [16,43,44,45,46,47,48]. According to the guidelines of the European Association for the Study of the Liver (EASL) and the Asian Pacific Association for the Study of the Liver (APASL), patients with advanced fibrosis or cirrhosis who have achieved SVR should be monitored for HCC every 6 months [45,49]. Patients without cirrhosis who achieved SVR according to the EASL guidelines can be considered definitively cured. Meanwhile, the APASL guidelines recommend monitoring such patients every 6 months for the first 2 years and then every 12 months, which is consistent with our observations [45,49].

The main limitation of the current analysis is the lack of an untreated control group. However, with safe and highly effective therapies available, it would be unethical to leave untreated patients with liver disease, especially advanced, for a long observation period. Other limitations of our study are related to the RWE design of the research, including its observational nature. The strongest point of our project is the longest follow-up of patients after effective DAA therapy to date. Data were collected from a real-world, heterogeneous population representative for routine practice. Additionally, the analyzed population was treated with one IFN-free regimen, which excludes the influence of the type of therapy on the obtained results. Finally, the percentage of patients lost to follow-up after 5 years was very small.

## 5. Conclusions

We confirmed that HCV clearance post-DAA therapy is durable regardless of baseline liver fibrosis. We found that persistent viral eradication is associated with a gradual but significant decrease over 5 years of liver stiffness. In contrast, liver function parameters improved during the initial 2 years of follow-up and then remained stable. The risk of HCC as well as HCV and HCC-related death persists throughout the 5-year observation after effective DAA treatment; however, in patients without cirrhosis, it seems to disappear 3 years after therapy, which requires confirmation during further follow-up. Monitoring for more than 5 years after curing HCV infection is necessary to assess the long-term risk of possible development of HCC, especially in patients with cirrhosis.

## Figures and Tables

**Table 1:** Baseline characteristics of patients available for evaluation after 5 years following end of treatment.

	*n* = 192
Age at the baseline of treatment (years), mean ± SD	54.3 ± 12.1
Males, *n* (%)	106 (55.2)
BMI (kg/m^2^), mean ± SD	25.9 ± 3.8
HCV genotype, *n* (%)	
1	9 (4.7)
1a	10 (5.2)
1b	164 (85.4)
4	9 (4.7)
History of previous therapy, *n* (%)	
treatment naïve	46 (24.0)
relapsers	33 (17.2)
partial responders	17 (8.9)
null responders	78 (40.6)
discontinued	4 (3.1)
unknown	14 (7.2)
Fibrosis, *n* (%)	
F0〓1	24 (12.5)
F2〓3	55 (28.6)
F4	110 (57.3)
unknown	3 (1.6)

BMI: Body mass index; HCV: Hepatitis C Virus.

**Table 2:** Medical events observed during 5 years of follow-up. Comparison of annual prevalence of events in the initial 2 years and following 3 years of follow-up. Fischer exact test.

Medical Event	between EOT and 2yFU *n* = 204		between 2yFU and 5yFU *n* = 192		*p*
	*n* EOT-2yFU (%)	*n* annual (%)	*n* 2yFU-5yFU (%)	*n* annual (%)	
Deaths	4 (2.0)	2.0 (1.0)	14 (7.3)	4.7 (2.4)	0.01
Deaths related to HCV	3 (1.5)	1.5 (0.7)	11 (5.7)	3.7 (1.9)	0.03
Ascites	11 (5.4)	5.5 (2.7)	6 (3.1)	2.0 (1.0)	0.32
Encephalopathy	5 (2.5)	2.5 (1.2)	0	0	0.06
Upper gastrointestinal bleadinng	3 (1.5)	1.5 (0.7)	1 (0.5)	0.3 (0.2)	0.62
Hepatocellular carcinoma	7 (3.4)	3.5 (1.7)	5 (2.6)	1.7 (0.9)	0.77
Liver transplantation	3 (1.5)	1.5 (0.7)	5 (2.6)	1.7 (0.9)	0.49

**Table 3:** Deaths in particular years of the follow-up and its relationship with HCV and hepatocellular carcinoma (HCC).

	Death	Year of the Follow-Up				
	Characteristics	1	2	3	4	5
baseline cirrhosis *n* = 110	not related to HCV or HCC	-	-	-	1	1
	related to HCV	-	1	2	3	1
	related to HCC	-	1	1	1	1
baseline no cirrhosis *n* = 79	not related to HCV or HCC	1	-	-	-	1
	related to HCV	-	-	1	-	-
	related to HCC	-	1	1	-	-

**Table 4:** Mean values of selected measures at the baseline, end of treatment (EOT), after 2 years follow-up (2yFU) and 5 years of follow-up (2yFU).

		Baseline		EOT			2yFU			5yFU		
	*n*	Mean	SD	Mean	SD	*p*	Mean	SD	*p*	Mean	SD	*p*
albumins, g/dL	137	4.05	0.53	4.22	0.50	<0.001	4.35	0.41	<0.001	4.35	0.50	0.39
bilirubin, g/dL	141	1.47	3.76	1.40	1.57	0.11	0.91	0.54	<0.001	0.93	0.50	0.13
MELD, score	121	8.66	2.67	8.54	2.59	0.74	7.75	1.90	<0.001	7.86	1.87	0.45
Child-Pugh, score	123	5.35	0.80	5.36	0.91	0.97	5.16	0.50	<0.001	5.11	0.41	0.19
INR	143	1.13	0.19	1.13	0.16	0.22	1.08	0.12	<0.001	1.07	0.14	0.05
BMI	129	25.9	3.77	25.9	3.75	0.39	26.3	3.99	<0.001	26.7	4.41	0.04
stiffness, kPa	78	17.9	13.0	15.4	11.4	<0.001	11.2	8.24	<0.001	9.65	5.59	0.001

*p* values refer to the comparison of the current and preceding time point (Wilcoxon matched paired test); MELD—Model for End-Stage Liver Disease, INR—International Normalized Ratio.

## Data Availability

Data are available upon request from the corresponding author.
